# Gemeinschaftsdiagnose: Die Krise wird allmählich überwunden

**DOI:** 10.1007/s10273-021-3026-y

**Published:** 2021-10-23

**Authors:** Martin Gornig, Oliver Holtemöller, Stefan Kooths, Torsten Schmidt, Timo Wollmershäuser

**Affiliations:** 1 Abtl. Industriepolitik, Deutsches Institut für Wirtschaftsforschung, Mohrenstr. 58, 10117 Berlin, Deutschland; 2 Abteilung Makroökonomik, Institut für Wirtschaftsforschung Halle, Kleine Märkerstraße 8, 06108 Halle (Saale), Deutschland; 3 Leiter Prognosezentrum, Institut für Weltwirtschaft, Kiellinie 66, 24105 Kiel, Deutschland; 4 RWI - Leibniz-Institut für Wirtschaftsforschung, Hohenzollernstraße 1-3, 45128 Essen, Deutschland; 5 und Befragungen, ifo Zentrum für Konjunkturforschung, Poschingerstraße 5, 81679 München, Deutschland

**Keywords:** E27, E32, E37

## Abstract

Die führenden Wirtschaftsforschungsinstitute senken ihre BIP-Wachstumsprognose für 2021 von 3,7 % auf 2,4 %. Dafür ist insbesondere die schwächelnde Industrieproduktion verantwortlich, die unter Lieferengpässen leidet. Die internationale Konjunktur erholt sich zwar von den Verwerfungen der Corona-Pandemie, aber nur langsam, da die Impffortschritte regional unterschiedlich sind. Die Verbraucherpreise haben sich 2021 stark erhöht.

## Abstract (en)

The leading economic research institutes have lowered their GDP growth forecast for 2021 from 3.7 % to 2.4 %. Weakening industrial production, which is suffering from supply bottlenecks, is particularly responsible for this. The global economy is recovering from the disruptions of the coronavirus pandemic, but only slowly, as vaccination progress varies across regions. Consumer prices increased sharply in 2021.

Die Pandemie wird in den nächsten Monaten die Konjunktur vor allem dort weiter belasten, wo die Impfquoten gering sind. Mit zunehmenden Quoten dürften sich die Rahmenbedingungen aber verbessern. Allerdings bremsen die Lieferengpässe, die sich wohl erst 2022 auflösen werden. So haben die Institute ihre Erwartung für den Zuwachs der Weltproduktion in diesem Jahr auf 5,7 % reduziert. Die Prognose für 2022 liegt bei 4,2 %. Der weltweite Warenhandel dürfte – trotz der schwachen Zunahme im Verlauf – im Durchschnitt des Jahres 2021 um 10,9 % zulegen. Für 2022 ist ein Anstieg um 3,1 % wahrscheinlich. Eines der Risiken bleibt die Möglichkeit neuer Virusvarianten. Ungewiss sind auch die finanzwirtschaftlichen Folgen der Pandemie. So bleibt abzuwarten, wie sich die Solvenz der Firmen entwickelt, wenn staatliche Kreditprogramme und Schuldenmoratorien auslaufen. Unklar sind derzeit auch die Folgen der finanziellen Probleme einzelner chinesischer Konglomerate.

Ein Unsicherheitsfaktor sind auch die von den Privathaushalten angehäuften Ersparnisse. Würden diese Polster für Konsumausgaben genutzt, ist zu vermuten, dass dies die Inflation weiter beschleunigt. Engpässe am Arbeitsmarkt zeichnen sich bereits ab, sie könnten zu stärkeren Lohnsteigerungen führen, als hier prognostiziert. Nicht nur die Verbraucherpreise, sondern auch die Preise für Vermögensgüter haben 2021 stark angezogen. Über die Preise für Rohstoffe als spezielle Form der Vermögensanlage pflanzt sich die Vermögenspreisinflation bereits auf Produktionskosten und Verbraucherpreise fort. Die inflationären Tendenzen könnten derart zunehmen, dass eine Straffung der Geldpolitik nötig wird.

## Die wirtschaftliche Lage in Deutschland

Nachdem neue Infektionswellen die Erholung in Deutschland im Winterhalbjahr 2020/2021 verzögert hatten, steigt das Bruttoinlandsprodukt (BIP) seit dem Abebben des Infektionsgeschehens im Frühjahr wieder deutlich. Allerdings behindern im Verarbeitenden Gewerbe Lieferengpässe bei Vorprodukten die Produktion, sodass bislang nur die von der Pandemie besonders betroffenen konsumnahen Dienstleistungsbranchen zulegen, während die Industrieproduktion seit Jahresbeginn sinkt. Für ihre Herbstprognose nehmen die Institute an, dass die wirtschaftliche Aktivität in Deutschland erst ab dem zweiten Quartal 2022 nicht mehr durch die Pandemie beeinträchtigt wird und dass sich die Lieferengpässe für Vorprodukte im Verlauf des Jahres 2022 auflösen

**Prof. Dr. Martin Gornig** ist Forschungsdirektor Industriepolitik und stellvertretender Leiter der Abteilung Unternehmen und Märkte am Deutschen Institut für Wirtschaftsforschung (DIW).

**Prof. Dr. Oliver Holtemöller** ist stellvertretender Präsident und leitet die Abteilung Makroökonomik am Leibniz-Institut für Wirtschaftsforschung Halle.

**Prof. Dr. Stefan Kooths** ist Direktor des Forschungszentrums Konjunktur und Wachstum am Institut für Weltwirtschaft in Kiel.

**Prof. Dr. Torsten Schmidt** ist Leiter des Kompetenzbereichs „Wachstum, Konjunktur, Öffentliche Finanzen" am RWI – Leibniz-Institut für Wirtschaftsforschung in Essen.

**Prof. Dr. Timo Wollmershäuser** ist stellvertretender Leiter des ifo Zentrums für Makroökonomik und Befragungen in München.

Tabelle 1
**Eckdaten der Prognose**

| | 2018 | 2019 | 2020 | 2021 | 2022 | 2023 |
|---|---|---|---|---|---|---|
| Reales BIP (Veränderung gegenüber dem Vorjahr in %) | 1,1 | 1,1 | -4,6 | 2,4 | 4,8 | 1,9 |
| Erwerbstätige im Inland in 1.000 Personen | 44.858 | 45.268 | 44.898 | 44.918 | 45.368 | 45.764 |
| Arbeitslose in 1.000 Personen | 2.340 | 2.267 | 2.695 | 2.636 | 2.448 | 2.356 |
| Arbeitslosenquote der Bundesagentur für Arbeit[1] in % | 5,2 | 5,0 | 5,9 | 5,7 | 5,3 | 5,1 |
| Verbraucherpreise[2] (Veränderung gegenüber dem Vorjahr in %) | 1,8 | 1,4 | 0,5 | 3,0 | 2,5 | 1,7 |
| Lohnstückkosten[3] (Veränderung gegenüber dem Vorjahr in %) | 3,0 | 3,1 | 3,4 | 0,8 | 0,0 | 1,8 |
| Finanzierungssaldo des Staats[4] | | | | | | |
| in Mrd. Euro | 64,4 | 51,1 | -145,2 | -173,7 | -80,5 | -35,1 |
| in % des nominalen BIP | 1,9 | 1,5 | -4,3 | -4,9 | -2,1 | -0,9 |
| Leistungsbilanzsaldo | | | | | | |
| in Mrd. Euro | 264,2 | 258,6 | 233,9 | 224,8 | 237,1 | 254,0 |
| in % des nominalen BIP | 7,8 | 7,4 | 6,9 | 6,4 | 6,3 | 6,5 |

[1] Arbeitslose in % der zivilen Erwerbspersonen (Definition gemäß der Bundesagentur für Arbeit). [2] Verbraucherpreisindex (2015 = 100). [3] Im Inland entstandene Arbeitnehmerentgelte je Arbeitnehmerstunde bezogen auf das reale BIP je Erwerbstätigenstunde. [4] In der Abgrenzung der Volkswirtschaftlichen Gesamtrechnungen (ESVG 2010).

Quellen: Statistisches Bundesamt; Bundesagentur für Arbeit; Deutsche Bundesbank; 2021 und 2023: Prognose der Institute. © GD Herbst 2021.

werden. Unter diesen Voraussetzungen dürfte die deutsche Wirtschaft im Sommer 2022 wieder normal ausgelastet sein; im Anschluss ist mit Nachholprozessen und damit einer zeitweisen Überauslastung zu rechnen. Insgesamt reduzieren die Institute ihre Prognose der Wachstumsrate des BIP für 2021 auf 2,4 % (Frühjahrs-Prognose: 3,7 %) und erhöhen sie für 2022 auf 4,8 % (Frühjahrs-Prognose: 3,9 %); 2023 dürfte das BIP um 1,9 % zulegen (vgl. Tabelle 1).

Die Finanzpolitik fährt mit dem Auslaufen von Corona-Hilfsmaßnahmen einen restriktiven Kurs. Angesichts der Regierungsneubildung besteht allerdings eine erhöhte Unsicherheit über die finanzpolitischen Maßnahmen. Eine Rückkehr zu einem ausgeglichenen Haushalt ist auch mit Erreichen der Normalauslastung vorerst nicht zu erwarten – nicht wegen der Pandemie, sondern aufgrund der in der vorangegangenen Legislaturperiode angelegten permanenten Ausgabensteigerungen. Das Defizit der öffentlichen Haushalte dürfte von 4,9 % in Relation zum BIP 2021 auf 2,1 % (2022) und 0,9 % (2023) zurückgehen. Wegen der kräftigen Zunahme des nominalen BIP wird die öffentliche Schuldenstandsquote wohl von 70,7 % (2021) auf 67,3 % (2022) und 64,9 % (2023) abnehmen. Gleichwohl ist das gesamtwirtschaftliche Umfeld für die Rückführung der Schuldenstandsquote mittelfristig ungünstiger als nach der Wirtschafts- und Finanzkrise.

## Öffentlichen Finanzen nach der Großen Rezession

Die Veränderung der Schuldenstandsquote lässt sich in die Komponenten nominales Wachstum, Finanzierungssaldo, Nettoerwerb von Finanzvermögen und übrige Einflussfaktoren zerlegen. Im Zuge der Finanzkrise war die Schuldenstandsquote 2008 bis 2010 auf 82 % gestiegen. Maßgeblich hierfür waren hohe Budgetdefizite und der bei der Einrichtung der Bad Banks erfolgte Erwerb von Finanzanlagen. Das Budgetdefizit 2010 war ebenfalls von den Bad Banks bestimmt, weil Wertverluste der von den Banken übernommenen Portfolios zu Buche schlugen. 2009 führte zudem das geringere nominale BIP zu einem Anstieg des relativen Bruttoschuldenstands. Den größten Beitrag – etwa drei Viertel – zur Reduktion der Schuldenstandsquote leistete das nominale BIP, das von 2010 bis 2019 mit einer durchschnittlichen Rate von 3,3 % zunahm. Dabei ging etwa die Hälfte des Anstiegs auf die Preisentwicklung zurück (vgl. Abbildung 1). Ab 2012 führten auch Überschüsse im Staatshaushalt zu rückläufigen Bruttoschulden. So sank die Schuldenstandsquote bis Ende 2019 unter den Maastricht-Referenzwert von 60 % – unter das Niveau von vor der Großen Rezession.

### Finanzierungsüberschüsse trotz expansiver Finanzpolitik

Der Finanzierungssaldo setzt sich aus einer konjunkturbereinigten und einer konjunkturabhängigen Komponente zusammen. Die Konjunkturkomponente ergibt sich dabei als Produkt der Budgetsemielastizität und der Produktionslücke. Der konjunkturbereinigte Finanzierungssaldo kann in einem weiteren Schritt in die Einmaleffekte, die diskretionären finanzpolitischen Maßnahmen, die progressionsbedingten Einnahmen sowie die Vermögenseinkommen zerlegt werden. Ausweislich der finanzpolitischen Maßnahmen war die Finanzpolitik in den vergangenen zehn Jahren außer 2011 und 2012 expansiv ausgerichtet (vgl. Abbildung 2). Während 2013, wie schon im Jahr zuvor, das Senken des Beitragssatzes zur gesetzlichen Rentenversicherung expansiv wirkte, wirkten in den Folgejahren kontinuierliche Leistungsausweitungen bei der gesetzlichen Rentenversicherung sowie

Abbildung 1

**Einflussfaktoren auf den Bruttoschuldenstand**

In Relation zum nominalen BIP in Prozentpunkten

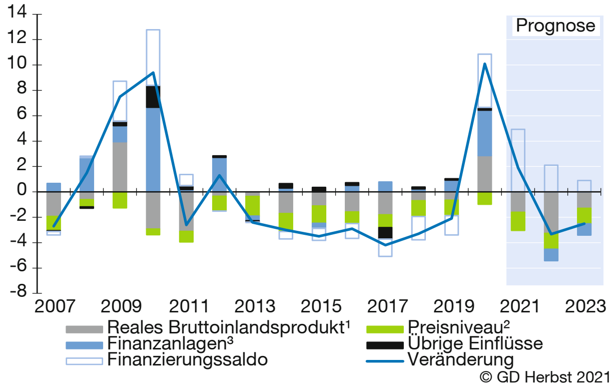

Anmerkung: [1] Einfluss der Veränderung des realen BIP auf die Schuldenstandsquote. [2] Einfluss der Veränderung des Deflators des BIP auf die Schuldenstandsquote. Für Preisniveau und reales BIP werden jeweils der „Shapley-Value" ausgewiesen, sodass die Reihenfolge der Dekomposition keinen Einfluss hat. [3] Nettoerwerb von Finanzanlagen.

Quellen: Datenbank AMECO der Europäischen Kommission; Eurostat; Berechnungen der Institute; 2021 bis 2023: Prognose der Institute. Zum „Shapley-Value": Shorrocks (2013).

Änderungen am Einkommensteuertarif expansiv. 2009 bis 2019 lag der Beitrag der finanzpolitischen Maßnahmen zur Veränderung des gesamtstaatlichen Finanzierungssaldos in Relation zum Produktionspotenzial durchschnittlich bei -0,3 Prozentpunkten. Es wurde also nicht aktiv konsolidiert, sondern in guten Jahren die finanzielle Ausgangslage für die kommenden Jahre verschlechtert. Die Produktionslücke war nach 2010 zumeist positiv, sodass die Konjunktur den Finanzierungssaldo anhob, und zwar mit einem durchschnittlichen Beitrag von 0,3 Prozentpunkten pro Jahr. Die Beiträge der Progression und der Vermögenseinkommen zur Veränderung des Finanzierungssaldos sind im Beobachtungszeitraum positiv, im Mittel liegen sie von 2010 bis 2019 bei 0,2 bzw. 0,1 Prozentpunkten. Dazu haben ein kräftiges Trendwachstum der Bruttolöhne und -gehälter sowie sinkende Kapitalmarktzinsen beigetragen.

## Strukturelle Dynamik des Arbeitsmarkts

Der Finanzierungssaldo lässt sich in einem weiteren Schritt anhand der einzelnen Komponenten des Staatskontos analysieren. Dabei werden die direkten und indirekten Steuern, die Sozialbeiträge, die arbeitsmarktbezogenen Ausgaben sowie die sonstigen Einnahmen und Ausgaben inklusive der Vermögenseinkommen um konjunkturelle Einflüsse und finanzpolitische Maßnahmen bereinigt. Anschließend wird die jahresdurchschnittliche Wachstumsdifferenz zwischen der so berechneten strukturellen Komponente und dem nominalen Produktionspotenzial über den Zeitraum zwischen 2010 und 2019 gebildet. Ohne strukturelle Sonderfaktoren sollten alle Komponenten mit der Rate des nominalen Produktions-

Abbildung 2

**Zerlegung der Veränderung des Finanzierungssaldos**

In Relation zum nominalen BIP in Prozentpunkten

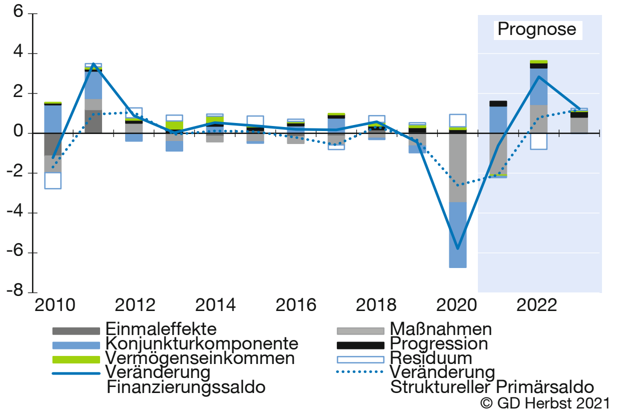

Quellen: Statistisches Bundesamt; Berechnungen der Institute; 2021 bis 2023: Prognose der Institute.

potenzials wachsen. Wenn hingegen die Einnahmekomponenten ein stärkeres Wachstum aufweisen als das nominale Produktionspotenzial, haben Mehreinnahmen strukturell zum Anstieg des Finanzierungssaldos beigetragen. Bei den Ausgabenkomponenten verhält es sich umgekehrt, hier bedeutet eine negative Differenz, dass die Minderausgaben strukturell zum Anstieg des Finanzierungssaldos im betrachteten Zeitraum beigetragen haben.

Zum Rückgang des strukturellen Defizits hat insbesondere die Entwicklung von Komponenten beigetragen, die von der positiven Entwicklung am Arbeitsmarkt profitierten (vgl. Abbildung 3). So nahmen zwischen 2010 und 2019 die Erwerbsbevölkerung und die Partizipationsquote stetig zu, gleichzeitig sank die strukturelle Erwerbslosenquote. Insgesamt stieg damit das trendmäßige Arbeitsvolumen um knapp 6 %. In der Folge nahmen die direkten Steuern, von denen ein wesentlicher Anteil die Lohnsteuer ausmacht, und die Sozialbeiträge um 1,2 bzw. 0,4 Prozentpunkte stärker zu als das nominale Produktionspotenzial. Die arbeitsmarktbezogenen Ausgaben, zu denen Transferzahlungen aus der Arbeitslosenversicherung zählen, wuchsen hingegen um 1,3 Prozentpunkte schwächer.

## Finanzpolitischer Ausblick für 2022 bis 2026

Die Schuldenstandsquote erreicht nach der Corona-Krise ein deutlich niedrigeres Niveau als nach der Finanzkrise, wobei Teilen der Schulden Finanzvermögen gegenübersteht, das in den kommenden Jahren wahrscheinlich aufgelöst wird und damit den Bruttoschuldenstand reduzieren dürfte. In der Summe könnte ausgehend vom Stand Ende 2020 die Reduktion des Finanzvermögens die Schuldenstandsquote bis 2026 um bis zu 5 Prozentpunkte mindern. Dagegen dürfte der Beitrag des nominalen BIP zur Rückführung der Schuldenstandsquote im Projektionszeitraum geringer ausfallen als in den Jahren nach der Wirtschafts- und Finanzkrise. Nach dem kräftigen Aufschwung 2022 und 2023 wird der reale Produktionszuwachs in Deutschland zunehmend durch Knappheiten auf dem Arbeitsmarkt begrenzt, weil die Zahl der Erwerbspersonen ab 2023 kontinuierlich zurückgeht. Der Deflator des BIP dürfte sich allerdings etwas dynamischer entwickeln als zuletzt. Ausgehend von einem Bruttoschuldenstand von 65 % 2023 bedeutete der in dieser Prognose unterstellte Zuwachs des nominalen BIP bei gleichbleibenden Bruttoschulden eine Reduktion der Schuldenstandsquote auf 60 % 2026.

2021 hebt die weiterhin expansive Finanzpolitik die positive Veränderung der Konjunkturkomponente auf; 2022 dürfte das Finanzierungsdefizit durch wegfallende coronabedingte Maßnahmen zurückgehen. In geringerem Umfang dürften beide Faktoren auch 2023 eine Rolle spielen. Die Steuerprogression dürfte in den nächsten Jahren selbst bei turnusgemäßem Ausgleich der kalten Progression weiter für steigende Einnahmen sorgen. Allerdings werden sich die strukturellen Veränderungen am Arbeitsmarkt, also der Rückgang der langfristigen Arbeitslosenquote, die steigende Partizipationsquote und die Zuwanderung nicht in dem Maße wie zuletzt fortsetzen. Die Erwerbsbevölkerung wird zurückgehen, die Erwerbslosenquote stagnieren und auch die Erwerbsbeteiligung ein Plateau erreichen. Somit dürfte das trendmäßige Arbeitsvolumen zwischen 2022 und 2026 um knapp 1 % sinken. Auch von den Zinsausgaben ist kein so hoher Beitrag zur Konsolidierung der öffentlichen Haushalte wie für die Vorjahre zu erwarten, hier besteht eher das Risiko steigender Zinsen. Die konjunkturelle Entwicklung trägt nur kurzfristig zum Abbau der Haushaltsdefizite bei. Schließlich dürfte der demografische Wandel den Sozialversicherungen zusetzen, was zu höheren Steuerzuschüssen führen wird.

## Wirtschaftspolitische Implikationen

Das Produktionspotenzial wird deutlich langsamer wachsen als bisher – zum einen, weil die Bevölkerung im erwerbsfähigen Alter zurückgeht, und zum anderen, weil Teile des gesamtwirtschaftlichen Kapitalstocks im Zuge der Dekarbonisierung obsolet werden. Die Institute rechnen mit einem Anstieg des Produktionspotenzials bis 2026 um durchschnittlich 1,0 % pro Jahr – bei abnehmenden Jahresraten zum Ende des Projektionszeitraums. Die Konsummöglichkeiten je Einwohner:in werden von zwei Seiten unter Druck geraten: Weniger Erwerbstätige je Einwohner müssen das Einkommen erwirtschaften, und ein größerer Teil des Einkommens muss investiert werden, um $CO_2$-Emissionen zu senken. Daher verringern sich die Konsummöglichkeiten, und nur durch gegenwärtigen Konsumverzicht wird es möglich sein, die avisierten Emissionsziele zu erreichen und die Staatsfinanzen nachhaltig aufzustellen. Wichtig ist, dass die Folgen von Demografie, Dekarbonisierung und Digitalisierung ungleich verteilt sind. Einkommensschwache Haushalte erwerben schon heute kaum auskömmliche Rentenansprüche und können Preissteigerungen durch Klimaschutzmaßnahmen nicht ohne weiteres tragen. Daher müssen die erforderlichen Maßnahmen sozial begleitet werden. Dies sollte vor allem durch direkte Hilfen für Bedürftige geschehen und nicht durch Verwässerung von Klimaschutzmaßnahmen. Der soziale Ausgleich ist aus der Verteilungsperspektive geboten. Er ist aber auch erforderlich für eine hohe Akzeptanz von technologischem Fortschritt, Strukturwandel und Klimaschutzpolitik.

Abbildung 3

**Wachstumsraten der Komponenten des Staatskontos**

Differenz zum nominalen Potenzialwachstum, 2010 bis 2019

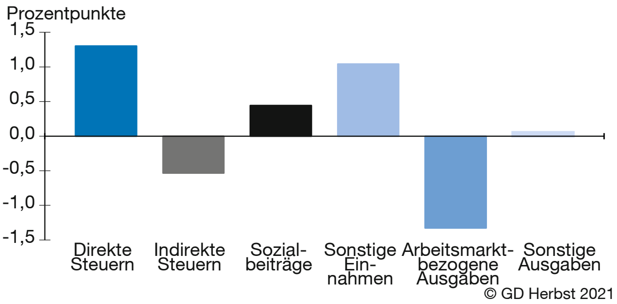

Quellen: Datenbank AMECO der Europäischen Kommission; Eurostat; Berechnungen der Institute.
